# Prevalence and Correlates of Undernutrition in Young Children Living in Urban Slums of Mumbai, India: A Cross Sectional Study

**DOI:** 10.3389/fpubh.2019.00191

**Published:** 2019-07-12

**Authors:** Samantha Lee Huey, Julia Leigh Finkelstein, Sudha Venkatramanan, Shobha A. Udipi, Padmini Ghugre, Varsha Thakker, Aparna Thorat, Ramesh D. Potdar, Harsha V. Chopra, Anura V. Kurpad, Jere Douglas Haas, Saurabh Mehta

**Affiliations:** ^1^Division of Nutritional Sciences, Cornell University, Ithaca, NY, United States; ^2^Institute for Nutritional Sciences, Global Health, and Technology (INSIGHT), Cornell University, Ithaca, NY, United States; ^3^Department of Nutrition and Food Science, SNDT Women's University, Mumbai, India; ^4^Centre for the Study of Social Change, Mumbai, India; ^5^Department of Physiology, St. John's Research Institute, Bangalore, India

**Keywords:** children, nutrition, Mumbai, anemia, stunting, India, growth, urban slums

## Abstract

**Background:** Young children living in urban slums are vulnerable to malnutrition and subsequently poor health outcomes, but data on the correlates of stunting, underweight, wasting, and anemia specifically among 10–18 month-old children in India remain limited.

**Objective:** In this analysis, we sought to describe the prevalence of and examine correlates for different markers of undernutrition, including stunting, underweight, and anemia among 10–18 month-old children living in urban slums, an understudied vulnerable group.

**Methods:** Children and their mothers (*n* = 323) were screened for anthropometry, demographics, and complete blood counts for hemoglobin concentration between March and November 2017 (Clinicaltrials.gov ID: NCT02233764). Correlates included child and mother's age, sex, birth order, birth weight, illness episodes, hemoglobin concentration, family income, maternal height, and maternal education level. Risk ratios (RR, 95% CI) for binary outcomes (stunting, underweight, wasting and anemia) and mean differences (β, 95% CI) for continuous outcomes (anthropometric Z-scores, hemoglobin concentration) were calculated using multivariate binomial and linear regression (SAS 9.4).

**Results:** The prevalence of stunting was 31.2%, underweight 25.1%, wasting (9.0%), and anemia (76%) among all children. Male children had a higher prevalence of poor growth indices and lower anthropometric Z-scores than females. Male sex, low birthweight, shorter maternal height, report of ≥1 episodes of illness within the past month, older maternal age, and birth order ≥2 were also associated with poor growth and anemia in multivariate models. Correlates of undernutrition were different among females and males. Female children had a 40% (20, 60%) higher risk of anemia associated with diarrhea, and male children who were firstborn had a 20% (0, 70%) lower risk of anemia.

**Conclusions:** These results show that poor growth and anemia among young children is prevalent in urban slums of Mumbai, and that sex of the child may play an important role in informing interventions to address undernutrition.

## Introduction

Describing the multifactorial determinants of undernutrition in young children living in urban slums—a high-risk group—in countries where undernutrition is highly prevalent such as India (1)—is of critical importance to identify and implement solutions that will enable the attainment of the current World Health Assembly Global Nutrition Targets for 2025 (2) and Sustainable Development Goals (particularly Goal 2) by 2030 (3).

Slum conditions are defined by the United Nations as having inadequate access to durable housing, a sufficient living area, safe water, sanitation, basic services, and protection against forced eviction (4–6). Globally, a total of 1 billion people reside in slums (7), including nearly half of Mumbai's population in 2011 (6). These slums are often unhealthy environments for populations as a whole and particularly for vulnerable groups such as young children (8–11). However, data on the prevalence and correlates for malnutrition are limited in this high-risk population, particularly in Indian urban slums (5, 8, 12, 13). As a recent review by Goudet et al. (8) argues, an improved understanding of the correlates for malnutrition in children in slums is needed to inform interventions, as well as provide guidance to practitioners and decision-makers. These correlates include both early-life determinants such as birth weight and maternal height/age, as well as current factors such as the child's age, family size, illness, and income level (8). Many studies have also identified sex differences in health and nutrition status: male children appear to have a higher prevalence of poor health outcomes than female children, both in terms of immune function (14) as well as metrics of poor growth (8).

There are several nutrition programs in India. For example, Integrated Child Development Services (ICDS) (15) which provides supplemental ration to 0–6 year-old children to improve nutrition status is run in urban slums of Mumbai; however, the prevalence of undernutrition in early childhood remains high (16). Therefore, the objective of this analysis was to describe and examine maternal and child correlates for markers of undernutrition, including poor growth and anemia among 10–18 month-old children living in urban slums of western Mumbai, India, an understudied vulnerable group. Additionally, this analysis will inform the selection and targeting of an appropriate population for a planned nutritional intervention of iron- and zinc-biofortified pearl millet (17, 18) in this setting (e.g., Clinicaltrials.gov ID: NCT02233764) (19) as well as for future nutritional intervention trials in other low-resource settings (e.g., Clinicaltrials.gov ID: NCT02648893).

## Materials and Methods

### Study Population, Setting, and Design

Eligibility for the current cross-sectional analysis was informed by a larger randomized controlled nutritional intervention efficacy trial (RCT) in which children aged between 10 and 18 completed months and their mothers living in 20 urban slum communities of Western Mumbai (including the East areas of Khar, Bandra, Santacruz) were screened for enrollment (Clinicaltrials.gov ID: NCT02233764; Clinical Trials Registry of India, CTRI: REF/2014/10/007731) (19). This parent RCT sought to follow up the participating children during their 2nd year of life (from 12 to 27 months of age), and therefore children were between 10 and 18 months old at screening prior to enrollment. The RCT protocol was reviewed and approved by the Inter Systems Biomedical Ethical Committee (ISBEC, Mumbai, Maharashtra, India), St. John's Research Institute (SJRI) Institutional Ethics Committee (IEC), and the Institutional Review Board (IRB) at Cornell University. In addition, permissions to conduct the study were obtained from the Health Ministry Screening Committee of India (Indian Council of Medical Research). Informed consent was obtained from all caregivers in an audio/visual format per Indian Government guidelines (20). Data included in the current analysis were collected from March to November 2017. After a census was conducted to identify children in the appropriate age group (10–18 months) residing in selected slums, caregivers were invited to come to the study center, Center for the Study of Social Change (CSSC, Bandra East, Mumbai), with their children, to be screened for eligibility in the randomized trial; inclusion criteria have been described previously (19). All children were provided 400 mg albendazole as recommended by World Health Organization (21) during screening under supervision by the study physician.

### Sample Size

The target sample size of the randomized trial was 96 participants, calculated by anticipated change in serum ferritin across 9 months of follow-up (19). The current analysis included all participants screened who had complete weight and other demographic data. For the current analysis, we determined that this sample was sufficient to achieve 81.3% power to detect a 1.5 higher risk ratio of stunting (our main outcome of interest) in male children (we hypothesized males were particularly vulnerable to undernutrition) when 31% of children were stunted overall.

### Assessment of Correlates

Data were collected from children at screening for the aforementioned randomized controlled trial, and therefore included data from children both eligible and ineligible to participate in the trial. Correlates included variables at the child level (age, sex, anthropometry, anemia and hemoglobin status, illness report), family level (income, birth order), and maternal level (age, height, education level) and are described below.

### Anthropometry

Trained research assistants collected anthropometric measurements using standardized procedures (22). The average of either duplicate (recumbent length, mid-upper-arm circumference, head circumference) and triplicate (triceps skinfold, subscapular skinfold) measurements was used as the final measurement. The weight of each child was measured using Rice Lake and Seca 703 body weight scales to the nearest 0.01 kg and calculated as the difference in weight of the child's caregiver alone compared to the weight of the caregiver holding the child, both wearing standard attire (without shoes) (GmbH & Co. KG, Hamburg, Germany). Maternal height was measured to the nearest 0.1 cm using an adult stadiometer (HeightLite, Weigh and Measure LLC, Olney, Maryland, USA). Child recumbent length was measured to the nearest 0.1 cm using an infant length board (ShorrBoard, Weigh and Measure LLC, Olney, Maryland, USA). Triceps skinfold and subscapular skinfold were measured to the nearest 5 mm (Lange Skinfold Caliper, Ann Arbor, MI, USA). Mid-upper-arm circumference (MUAC) and head circumference were measured to the nearest 0.1 cm (Shorr Child MUAC Weigh and Measure LLC, Olney, Maryland, USA). Maternal height [continuous (cm) or categorized as short stature defined as <150 cm (23)] was included as a correlate.

### Demographic and Health History

Research assistants collected maternal and child demographic and health history data through interviews with caregivers. Sociodemographic factors included age of the child (age was determined by caregiver's recall and confirmed by maternal/child health card), sex of the child, child's birth weight low birthweight [defined as <2.5 kg (24, 25), was determined by caregiver's recall and confirmed by maternal/child health card)], maternal age, highest level of education for the mother [less education was defined as less than and including 8 years or eighth standard, at which point it is common for students from slum areas to stop going to school (26, 27)] low income [defined as <10,000 Indian Rupees (INR) per month, which is equivalent to 142% of the currently recommended poverty line of 7,035 INR/month (approx. USD 110 at the time of the study) for a family of 5 living in an urban setting (28)] and reported birth order (first-born/only child compared to later-born). The child's health history data was reported by the mother (including if the child having had any occurrence of diarrhea, fever, cough, or other illness within the past month) and a physical examination was conducted by the study clinician.

### Biological Samples

At the study center (CSSC), a pediatric phlebotomist applied topical anesthetic [Prilox Cream (lidocaine with prilocaine), Neon Laboratories Limited, Mumbai, India], topical antisepsis and then collected venous blood from the antecubital vein. After centrifugation to separate serum from whole blood, blood was divided into aliquots and immediately transported (within a range of 1–6 h after collection) to SRL Diagnostics (Goregaon, Mumbai) for immediate analysis as well as storage at −80°C for future batch analyses of nutrition status and immune function. Complete blood counts including hemoglobin, hematocrit (Hct), mean cell volume (MCV), and mean corpuscular hemoglobin (MCH) were immediately analyzed by SRL Diagnostics laboratory in Mumbai (Beckman Coulter Counter). Anemia was defined as hemoglobin concentration <11.0 g/dL (29). Microcytic hypochromic anemia was defined as the presence of anemia with MCV <77 fL (30) and MCH <34.4 pg (29, 31).

### Assessment of Outcome and Outcome Definitions

Outcome variables included stunting, underweight, wasting, length-for-age Z-score (LAZ), weight-for-age Z-score (WAZ), weight-for-length Z-score (WLZ), anemia, and hemoglobin concentration among children. Anthropometric Z scores, continuous or categorized as <2 standard deviations (SDs) from the reference standard median, were calculated using WHO International Growth References (version 3.2.2, 2011, http://www.who.int/childgrowth/en/). Underweight was defined as WAZ <2 SDs, stunting as LAZ <2 SDs, and wasting as WLZ < -2 SDs below the reference standard (32). Continuous hemoglobin concentration and hemoglobin concentration below 11.0 g/dL (i.e., anemia) were also analyzed as outcomes (29).

### Statistical Analysis

Continuous variables were analyzed for normality using the Shapiro–Wilk test; if data were not normally distributed, means and interquartile ranges (IQR) were reported. Hodges-Lehmann-Sen tests were used to compare continuous variables between groups. Categorical data were compared using the Chi Square test and Fisher's Exact test where counts per cell were less than five. Risk ratios (RRs) and 95% confidence intervals (CI) were estimated by binomial regression with the log-link function. If the model failed to converge, a log-Poisson regression approach was used to estimate the RR and 95% CI (33). Potential correlates were known or probable risk factors for the outcome based on biological plausibility and previous literature (8). Correlates associated with the outcome of interest at *P* < 0.20 in univariate analysis were included in the multivariate model; only those correlates were retained in the model that were associated with the outcome with a *P-value* of ≤ 0.05 (34). Analyses were also conducted separately for male and for female children. We used the missing indicator method to account for missingness in correlates. All analyses were two-sided and differences between groups were considered significant at *P* < 0.05. Data were analyzed using SAS version 9.4 (SAS Institute, Cary, North Carolina, USA). These data were subject to cross-checking and confirmation by the Cornell Institute for Social and Economic Research (CISER) to ensure reproducibility.

## Results

### Characteristics of Study Participants

A total of 407 children residing in 20 urban slums of Western Mumbai were screened for eligibility into the parent trial between March and November 2017. From the time period of screening, both anthropometry and sociodemographic data from 323 children, including 277 children with adequate whole blood collected for complete blood counts, were available for analyses.

Characteristics of the population are presented in Table 1. Male and female children were similar in most characteristics, except that males were heavier and had a higher head circumference compared to female children. Low birthweight was reported by mothers in 20.7% of children. Report of at least 1 episode of an illness (diarrhea, cough, and/or fever) were reported to have affected 11.4–37.1% of participants within the past month. Nearly half of the children were the first-born child in the family. Over 70% of mothers reported their maximum level of education attained was ≤8th standard.

**Table 1:** Characteristics of cohort (*N* = 323).

**Characteristic**	***N***	***n* (%) or median (IQR)**	**Males (*n* = 164)** ***n* (%) or median (IQR*)***	**Females (*n* = 159)** ***n* (%) or median (IQR)**	***P*-value**
Child's age (mo)	323	14.5 (12.7, 16.7)	14.4 (12.5, 16.4)	14.6 (13.0, 16.9)	0.12
Age <15 mo	323	184 (57.0)	99/164 (60.4)	85/159 (53.5)	0.21
Birth weight (kg)	323	2.7 (2.5, 3.0)	2.6 (2.5, 3.0)	2.8 (2.5, 3.0)	0.54
Low birthweight (<2.5 kg)		67 (20.7)	34/164 (20.7)	33/159 (20.8)	0.99
Weight (kg)	323	8.6 (7.9, 9.4)	8.7 (8.0, 9.6)	8.3 (7.8, 9.1)	**<0.01**
Length (cm)	321	74.2 (72.0, 77.0)	74.2 (72.3, 77.0)	74.0 (71.8, 77.0)	0.12
Head circumference (cm)	322	44.3 (43.3, 45.3)	44.8 (43.8, 45.9)	43.6 (42.9, 44.6)	**<0.0001**
Mid-upper-arm circumference (cm)	323	14.3 (13.6, 14.9)	14.4 (13.7, 15.0)	14.2 (13.5, 14.8)	0.12
Triceps skinfold (mm)	322	7.67 (6.67, 9.00)	7.85 (6.67, 9.25)	7.50 (6.50, 8.83)	0.15
Subscapular skinfold (mm)	322	7.00 (6.03, 8.33)	7.00 (6.17, 8.33)	6.83 (6.00, 8.00)	0.42
Length-for-age Z-score	321	−1.40 (−2.14, −0.64)	−1.67 (−2.32, −0.69)	−1.19 (−1.97, −0.54)	**0.01**
Stunting		100 (31.2)	62/162 (38.3)	38/159 (23.9)	**<0.01**
Severe stunting		26 (8.1)	16/162 (9.9)	10/159 (6.3)	0.24
Weight-for-age Z-score	323	−1.29 (−2.01, −0.52)	−1.41 (−2.13, −0.56)	−1.13 (−1.77, −0.45)	**<0.05**
Underweight		81 (25.1)	49/164 (29.9)	32/159 (20.1)	**<0.05**
Severe underweight		11 (3.4)	8/164 (4.9)	3/159 (1.9)	0.22^a^
Weight-for- length Z-score	321	−0.77 (−1.40, −0.21)	−0.84 (−1.56, −0.24)	−0.72 (−1.29, −0.14)	0.10
Wasting		29 (9.0)	20/162 (12.4)	9/159 (5.7)	**<0.05**
Severe wasting		4 (1.25)	2/162 (1.2)	2/159 (1.3)	0.99^a^
Stunting, underweight, or wasting [CIAF (33, 34)]	321	122 (38.0)	76/162 (46.9)	46/159 (28.9)	**<0.001**
Stunted and underweight	321	63 (19.6)	37/162 (22.8)	26/159 (16.4)	0.14
Wasted and underweight	321	25 (7.8)	18/162 (11.1)	7/159 (4.4)	**<0.05**
Stunted and wasted and underweight	321	16 (5.0)	11/162 (6.8)	5/159 (3.1)	0.13
Hemoglobin (g/dL)	277	10.1 (8.9, 10.9)	9.8 (8.7, 10.9)	10.2 (9.3, 11.0)	0.059
Anemia (Hb <11.0 g/dL)		209 (75.5)	110/142 (77.5)	99/135 (73.3)	0.42
Severe Anemia (Hb <7.0 g/dL)		8 (2.9)	5/142 (3.5)	3/135 (2.2)	0.72^a^
Microcytic hypochromic anemia		206 (74.4)	109/142 (76.8)	97/135 (71.9)	0.35
Diarrhea today or within past mo	299	34 (11.4)	14/151 (9.3)	20/148 (13.5)	0.25
Cough today or within past mo	299	95 (31.8)	52/151 (34.4)	43/148 (29.1)	0.32
Fever today or within past mo	299	111 (37.1)	60/151 (39.7)	51/148 (34.5)	0.35
Exclusive breastfeeding ≥6 mo	320	218 (68.1)	109/161 (67.7)	109/159 (68.6)	0.87
First-born in family	319	135 (42.3)	73/161 (45.3)	62/158 (39.2)	0.27
Income <10,000 INR per mo	321	88 (27.4)	118/162 (72.8)	115/159 (72.3)	0.92
Maternal height (cm)	323	150.5 (147.0, 154.5)	150.0 (146.1, 154.0)	151.5 (147.6, 155.0)	0.13
Maternal short stature (height <150 cm)	323	138 (42.7)	76/164 (46.3)	62/159 (39.0)	0.18
Maternal age (years)	320	27 (25, 30)	27 (25, 30)	27 (24, 30)	0.93
Maternal education: ≤8th standard	318	231 (72.6)	117/160 (73.1)	114/158 (72.2)	0.85
Paternal education: ≤8th standard	319	260 (81.5)	126/160 (78.8)	134/159 (84.3)	0.20

*Correlates refer to child unless noted by “maternal”; 14% of blood data are missing. Microcytic hypochromic anemia is defined as MCV <77 fL (30), MCH <34.4 pg (31), and hemoglobin <11 g/dL (29). LAZ, length-for-age Z-score; WAZ, weight-for-age Z-score; WLZ, weight-for-length Z-score; underweight, WAZ < -2; severe underweight, WAZ < -3; stunting, LAZ < -2; severe stunting, LAZ < -3; wasting, WLZ < -2; severe wasting, WLZ < -3;CIAF, comprehensive index of anthropometric failure; low income defined as <10,000 Indian Rupees (INR) per month (142% of the poverty line) (28); cough, fever, and diarrhea as reported occurring today or within the past 4 weeks. P-values represent Chi-Square or Hodges-Lehmann-Sen tests as appropriate*.

a *Fisher's Exact test was performed to generate this P-value. Bolded values mean statistical significance*.

### Correlates of Poor Growth and Anemia

The median (IQR) LAZ, WAZ, and WLZ across the sample was −1.40 (−2.14, −0.64), −1.29 (−2.01, −0.52), and −0.77 (−1.40, −0.21), respectively. The prevalence of stunting, underweight, and wasting was 31.2, 25.1, and 9.0%, and each was more common among males than females (Table 1). The median (IQR) hemoglobin concentration was 10.1 (8.9, 10.9) g/dL, and over 75% of children were anemic (hemoglobin <11 g/dL).

In multivariate models, male sex was associated with a 50% (95% CI: 10, 110%) higher risk of stunting and lower LAZ score [−0.30 (95% CI: −0.54, −0.07)] (Table 2). Other correlates of stunting in the cohort included low birthweight [RR: 1.4 (95% CI: 1.0, 1.8)] and maternal height, which was associated with a 7% (95% CI: 5, 9%) lower risk of stunting per every additional centimeter. Low birthweight, short maternal stature, and maternal education ≤8th standard were all significantly associated with 0.39–0.46 lower LAZ. Among male children, increasing maternal height was protective against stunting and associated with higher LAZ, in addition to advanced maternal education [−0.40 (95% CI: −0.78, −0.02)] (Table 3). In female children, report of cough in the past 1 month was associated with an over 2-fold higher risk of stunting [RR: 2.2 (95% CI: 1.3, 3.6)] (Table 4). Increasing maternal height was also associated lowered risk of stunting in female children as well as higher LAZ (Table 4). Other factors that were associated with lower LAZ in female children included low birthweight [−0.69 (95% CI: −1.08, −0.31)] and less maternal education [−0.41 (95% CI: −0.75, −0.06)]. Fever was also associated with higher LAZ in female children [0.53 (0.19, 0.88)].

**Table 2:** Correlates of poor growth and anemia among *n* = 323 children.

**Correlate** **(univariate,** ** multivariate)**	**Continuous and dichotomous outcomes (Unadjusted univariate and adjusted multivariate analysis)**							
	**LAZ β (95% CI)**	**Stunting RR (95% CI)**	**WAZ β (95% CI)**	**Underweight RR (95% CI)**	**WLZ β (95% CI)**	**Wasting RR (95% CI)**	**Hemoglobin (g/dL) β (95% CI)**	**Anemia RR (95% CI)**
Age (days)	−0.00 (−0.00, 0.00)	1.0 (−1.0, 1.0)	−0.00 (−0.00, 0.00)	1.0 (1.0, 1.0)	1.00 (1.00, 1.00)	1.0 (−0.0, 0.0)	0.00 (−0.00, 0.00)	1.0 (1.0, 1.0)
Age as factor in multivariate model								
Male (vs. female)	−0.32 (−0.57, −0.07)^**^	1.6 (1.1, 2.2)^**^	−0.25 (−0.48, −0.01)^**^	1.5 (1.0, 2.2)^**^	−0.16 (−0.38, 0.06)^*^	2.2 (1.0, 4.6)^**^	−0.36 (−0.70, −0.01)^**^	1.1 (0.9, 1.2)
Male as factor in multivariate model	−0.30 (−0.54, −0.07)^**^	1.5 (1.1, 2.1)^**^	−0.22 (−0.44, −0.00)^**^	1.4 (1.0, 2.0)	−0.13 (−0.35, 0.08)	2.1 (0.9, 4.5)	−0.44 (−0.77, −0.11)^**^	
Low birthweight (vs. birthweight >2.5 kg)	−0.60 (−0.90, −0.29)^**^	1.6 (1.2, 2.3)^**^	−0.81 (−1.09, −0.53)^***^	1.9 (1.3, 2.8)^**^	−0.66 (−0.93, −0.39)^***^	3.1 (1.6, 6.1)^**^	−0.51 (−0.94, −0.08)^**^	1.1 (1.0, 1.3)^*^
Low birthweight as factor in multivariate model	−0.46 (−0.76, −0.17)^**^	1.4 (1.0, 1.8)^**^	−0.69 (−0.96, −0.42)^***^	1.7 (1.2, 2.5)^**^	−0.62 (−0.88, −0.35)^***^	2.9 (1.5, 5.6)^**^	−0.50 (−0.90, −0.09)^**^	1.1 (0.8, 1.6)
Hemoglobin (g/dL)	0.10 (0.00, 0.19)^**^	0.9 (0.8, 1.0)^**^	0.06 (−0.03, 0.15)^*^	1.0 (0.9, 1.2)	0.01 (−0.07, 0.09)	1.0 (0.8, 1.3)	n/a	n/a
Hemoglobin as factor in multivariate model	0.05 (−0.04, 0.14)	1.0 (0.8, 1.1)	0.00 (−0.08, 0.08)				n/a	n/a
Anemia (vs. hemoglobin >11 g/dL)	−0.10 (−0.43, 0.22)	1.1 (0.7, 1.7)	1.01 (0.75, 1.37)	0.9 (0.5, 1.3)	0.14 (−0.15, 0.42)	0.9 (0.4, 2.3)	n/a	n/a
Anemia as factor in multivariate model							n/a	n/a
Diarrhea [vs. no diarrhea episode(s) in the past month]	0.29 (−0.12, 0.69)^*^	0.7 (0.4, 1.4)	1.26 (0.86, 1.86)	0.9 (0.5, 1.8)	0.13 (−0.23, 0.50)	0.7 (0.2, 2.7)	−0.81 (−1.37, −0.25)^**^	1.3 (1.2, 1.5)^***^
Diarrhea as factor in multivariate model	0.25 (−0.14, 0.63)						−0.69 (−1.23, −0.16)^**^	1.3 (1.2, 1.5)^***^^%^
Cough [vs. no cough episode(s) in the past month]	0.87 (0.66, 1.14)	1.3 (0.9, 1.8)^*^	−0.23 (−0.50, 0.03)^*^	1.4 (0.9, 2.1)^*^	−0.19 (−0.44, 0.06)^*^	2.0 (0.9, 4.1)^*^	−0.07 (−0.45, 0.31)	0.9 (0.8, 1.1)
Cough as factor in multivariate model		1.1 (0.8, 1.6)	−0.19 (−0.43, 0.05)	1.1 (0.7, 1.7)	−0.18 (−0.42, 0.06)	1.4 (0.6, 3.2)		
Fever [vs. no fever episode(s) in the past month]	0.19 (−0.08, 0.46)^*^	1.0 (0.7, 1.5)	0.00 (−0.25, 0.25)	1.4 (0.9, 2.0)^*^	−0.14 (−0.38, 0.09)	3.0 (1.4, 6.6)^**^	0.11 (−0.26, 0.48)	0.9 (0.8, 1.1)^*^
Fever as factor in multivariate model	0.24 (−0.02, 0.49)			1.3 (0.9, 1.9)		2.8 (1.3, 6.1)^**^		0.9 (0.7, 1.2)
First born (vs. later born)	0.07 (−0.19, 0.32)	1.1 (0.8, 1.5)	0.12 (−0.12, 0.36)	1.0 (0.7, 1.4)	0.12 (−0.11, 0.34)	0.9 (0.4, 1.8)	0.39 (0.03, 0.74)^**^	0.8 (0.7, 1.0)^**^
First born as factor in multivariate model							0.56 (0.21, 0.92)^**^	0.9 (0.6, 1.1)
Low income (vs. >10,000 rupees/month)	−0.31 (−0.59, −0.03)^**^	1.3 (0.9, 1.8)^*^	−0.27 (−0.53, −0.00)^**^	1.2 (0.8, 1.8)	−0.13 (−0.38, 0.12)	1.1 (0.5, 2.3)	−0.35 (−0.74, 0.03)^*^	1.0 (0.9, 1.2)
Income as factor in multivariate model	1.0 (0.7, 1.3)	1.0 (0.6, 1.6)	−0.02 −0.27, 0.24)				−0.27 (−0.64, 0.10)	
Maternal age (y)	−0.00 (−0.03, 0.03)	1.0 (−1.0, 1.1)	0.01 (−0.02, 0.03)	1.0 1.0, 1.1)	1.01 (0.98, 1.04)	1.1 (1.0, 1.15)^*^	0.05 (0.01, 0.09)^**^	1.0 (1.0, 1.0)
Maternal age as factor in multivariate model						1.1 (1.0, 1.2)	0.07 (0.03, 0.11)^**^	
Maternal height (cm)	0.06 (0.04, 0.08)^***^	0.9 (0.9, 1.0)^***^	0.05 (0.03, 0.07)^***^	0.9 (0.9, 1.0)^***^	0.03 (0.01, 0.05)^**^	0.9 (0. 9, 1.0)^*^	0.02 (−0.01, 0.05)^*^	1.0 (1.0, 1.0)
Maternal height as factor in multivariate model	0.06 (0.03, 0.10)	0.9 (0.9, 1.0)^***^	0.04 (0.02, 0.06)^***^	0.9 (0.9, 1.0)^**^	0.02 (0.002, 0.04)^**^	1.0 (0.9, 1.0)	0.02 (−0.01, 0.04)	
Maternal height <150 cm (vs. ≥150 cm)	−0.57 (−0.82, −0.32)^***^	2.2 (1.6, 3.1)^***^	−0.50 (−0.73, −0.26)^***^	2.1 (1.4, 3.0)^**^	−0.28 (−0.50, −0.05)^**^	1.9 (0.9, 3.9)^*^	−0.30 (−0.64, 0.05)^*^	1.1 (0.9, 1.2)
Short maternal height as factor in multivariate model	−0.45, −0.69, −0.21)^**^	1.1 (0.6, 2.2)	0.12 (−0.23, 0.48)	1.1 (0.5, 2.1)	0.06 (−0.30, 0.13)	0.9 (0.8, 1.0)	−0.10 (−0.63, 0.43)	
Maternal education ≤8th standard (vs. >8th)	−0.48 (−0.76, −0.20)^**^	1.3 (1.0, 1.9)^*^	−0.38 (−0.64, −0.12)^**^	1.5 (1.0, 2.2)^**^	−0.20 (−0.45, 0.05)^*^	0.9 (0.4, 2.0)	−0.11 (−0.49, 0.28)	1.0 (0.8, 1.1)
Maternal education as factor in multivariate model	−0.39 (−0.66, −0.12)^**^	1.1 (0.8, 1.5)	−0.27 (−0.52, −0.02)^**^	1.3 (0.9, 1.8)	−0.11 (−0.36, 0.13)			

*Correlates refer to child unless noted by “maternal”; LAZ, length-for-age Z-score; WAZ, weight-for-age Z-score; WLZ, weight-for-length Z-score; low income defined as <10,000 Indian Rupees (INR) per month (142% of the poverty line) (28); cough, fever, and diarrhea as reported occurring today or within the past 4 weeks*.

* *P ≤ 0.20*;

** *P <0.05*;

*** *P <0.0001*;

% *Multivariate model retained only 1 correlate signifcant at p <0.05*.

**Table 3:** Correlates of poor growth and anemia among *n* = 164 male children.

**Correlate** **(univariate,** ** multivariate)**	**Continuous and dichotomous outcomes (Unadjusted univariate and adjusted multivariate analysis)**					
	**LAZ β (95% CI)**	**Stunting RR (95% CI)**	**WAZ β (95% CI)**	**Underweight RR (95% CI)**	**Hemoglobin (g/dL) β (95% CI)**	**Anemia RR (95% CI)**
Age (days)	0.00 (−0.00, 0.00)	1.0 (1.0, 1.0)	1.00 (1.00, 1.00)	1.0 (1.0, 1.0)	1.00 (1.00, 1.01)	1.0 (1.0, 1.0)
**Age as factor in multivariate model**						
Low birthweight (vs. birthweight >2.5 kg)	−0.35 (−0.79, 0.09)^*^	1.3 (0.9, 2.0)	−0.61 (−1.03, −0.19)^**^	1.5 (0.9, 2.5)^*^	−0.55 (−1.18, 0.07)^*^	1.2 (1.0, 1.4)^*^
Low birthweight as factor in multivariate model	−0.27 (−0.68, 0.14)		−0.54 (−0.95, −0.13)^**^	1.3 (0.8, 2.0)	0.6 (0.3, 1.1)	1.1 (0.7, 1.8)
Hemoglobin (g/dL)	0.07 (−0.05, 0.19)	0.9 (0.8, 1.1)	1.03 (0.91, 1.16)	1.1 (0.9, 1.3)	n/a	n/a
Hemoglobin as factor in multivariate model					n/a	n/a
Anemia (vs. hemoglobin >11 g/dL)	−0.19 (−0.66, 0.28)	1.0 (0.6, 1.6)	0.09 (−0.36, 0.53)	0.7 (0.4, 1.2)	n/a	n/a
Anemia as factor in multivariate model					n/a	n/a
Diarrhea [vs. no diarrhea episode(s) in the past month]	0.24 (−0.41, 0.89)	0.7 (0.3, 1.7)	0.41 (−0.22, 1.03)	1.0 (0.4, 2.3)	−0.94 (−1.84, −0.05)^**^	1.3 (na)^$^
Diarrhea as factor in multivariate model					0.56 (0.06, 1.07)	
Cough (vs. no cough episode(s) in the past month)	−0.01 (−0.40, 0.39)	0.9 (0.6, 1.4)	−0.08 (0.47, 0.30)	1.2 (0.7, 1.9)	−0.03 (−0.58, 0.52)	0.9 (0.8, 1.2)
Cough as factor in multivariate model						
Fever [vs. no fever episode(s) in the past month]	0.06 (−0.32, 0.45)	1.2 (0.8, 1.9)	−0.13 (−0.51, 0.24)	1.6 (1.0, 2.6)^*^	0.13 (−0.41, 0.68)	0.9 (0.7, 1.1)
Fever as factor in multivariate model				1.4 (0.9, 2.3)		
First born (vs. later born)	0.17 (−0.20, 0.53)	1.0 (0.6, 1.4)	0.30 (−0.05, 0.64)^*^	0.9 (0.5, 1.4)	0.62 (0.12, 1.13)^**^	0.9 (0.7, 1.0)^*^
First born as factor in multivariate model			0.21 (−0.13, 0.54)		0.62 (0.12, 1.13)^**^^%^	0.8 (0.7, 1.0)^**^
Low income (vs. >10,000 rupees/month)	−0.11 (−0.52, 0.29)	1.1 (0.7, 1.7)	−0.23 (−0.62, 0.16)	1.1 (0.6, 1.9)	−0.34 (−0.92, 0.23)	1.0 (0.8, 1.2)
Income as factor in multivariate model						
Maternal age (y)	−0.04 (−0.09, 0.01)^*^	1.0 (1.0, 1.1)^*^	0.97 (0.93, 1.01)	1.01 (0.95, 1.08	0.02 (−0.05, 0.10)	1.0 (1.0, 1.0)^*^
Maternal age as factor in multivariate model	−0.02 (−0.06, 0.03)	1.0 (0.9, 1.1)				1.0 (1.0, 1.0)^**^
Maternal height (cm)	0.07 (0.04, 0.10)^***^	0.9 (0.9, 1.0)^**^^$^	0.06 (0.03, 0.09)^**^	0.9 (0.9, 1.0)^**^	−0.01 (−0.05, 0.04)	1.0 (1.0, 1.0)
Maternal height as factor in multivariate model	0.07 (0.04, 0.10)^***^	0.9 (0.9, 1.0)^**^^$^^%^	0.05 (0.03, 0.08)^**^	0.9 (0.9, 1.0)^**^^%^		
Maternal height <150 cm (vs. ≥150 cm)	−0.52 (−0.87, −0.16)^**^	2.0 (1.3, 3.0)^**^	−0.44 (−0.79, −0.10)^**^	1.8 (1.1, 3.0)^**^	−0.24 (−0.76, 0.27)	1.0 (0.8, 1.2)
Short maternal height as factor in multivariate model	0.34 (−0.19, 0.88)	1.2 (0.5, 2.7)	0.29 (−0.24, 0.81)	0.8 (0.3, 1.9)		
Maternal education ≤8th standard (vs. >8th)	−0.44 (−0.85, −0.03)^**^	1.2 (0.8, 1.8)	−0.28 (−0.67, 0.11)^*^	1.1 (0.7, 1.9)	−0.08 (−0.64, 0.48)	1.0 (0.8, 1.2)
Maternal education as factor in multivariate model	−0.40 (−0.78, −0.02)^**^		−0.24 (−0.61, 0.14)			

*Correlates refer to child unless noted by “maternal”; LAZ, length-for-age Z-score; WAZ, weight-for-age Z-score; WLZ, weight-for-length Z-score; low income defined as <10,000 Indian Rupees (INR) per month (142% of the poverty line) (28); cough, fever, and diarrhea as reported occurring today or within the past 4 weeks*.

* *P ≤ 0.20*;

** *P <0.05*;

*** *P <0.0001*;

$ *Poisson distribution used instead of binomial regression due to non-convergence*;

% *Multivariate model retained only 1 correlate signifcant at p <0.05*.

**Table 4:** Correlates of poor growth and anemia among *n* = 159 female children.

**Correlate** ** (univariate,** ** multivariate)**	**Continuous and dichotomous outcomes (Unadjusted univariate and adjusted multivariate analysis)**					
	**LAZ β (95% CI)**	**Stunting RR (95% CI)**	**WAZ β (95% CI)**	**Underweight RR (95% CI)**	**Hemoglobin (g/dL) β (95% CI)**	**Anemia RR (95% CI)**
Age (days)	−0.00 (−0.00, 0.00)^*^	1.0 (1.0, 1.0)	−0.00 (−0.00, 0.00)	1.0 (1.0, 1.0)	−0.00 (−0.00, 0.00)	1.0 (1.0, 1.0)
Age as factor in multivariate model	−0.00 (−0.00, 0.00)					
Low birthweight (vs. birthweight >2.5 kg)	−0.85 (−1.25, −0.44)^***^	2.2 (1.3, 3.8)^**^	−1.01 (−1.37, −0.65)^***^	2.6 (1.4, 4.7)^**^	−0.44 (−1.01, 0.13)^*^	1.1 (0.8, 1.3)
Low birthweight as factor in multivariate model	−0.69 (−1.08, −0.31)^**^	1.7 (0.9, 3.4)	−0.86 (−1.22, −0.51)^***^	2.2 (1.2, 4.0) ^**^	−0.28 (−0.84, 0.28)	
Hemoglobin (g/dL)	0.10 (−0.04, 0.24)^*^	0.9 (0.7, 1.0)^*^	0.08 (−0.06, 0.21)	1.0 (0.8, 1.2)	n/a	n/a
Hemoglobin as factor in multivariate model	0.01 (−0.12, 0.14)	0.9 (0.7, 1.2)			n/a	n/a
Anemia (vs. hemoglobin >11 g/dL)	0.02 (−0.42, 0.46)	1.2 (0.6, 2.4)	−0.02 (−0.43, 0.38)	1.0 (0.5, 2.2)	n/a	n/a
Anemia as factor in multivariate model					n/a	n/a
Diarrhea [vs. no diarrhea episode(s) in the past month]	0.27 (−0.24, 0.78)	0.8 (0.3, 2.0)	0.07 (−0.41, 0.54)	1.0 (0.4, 2.6)	−0.78 (−1.46, −0.10)^**^	1.4 (1.2, 1.6)^**^
Diarrhea as factor in multivariate model					−0.70 (−1.35, −0.05)^**^	1.4 (1.2, 1.6)^**^^%^
Cough [vs. no cough episode(s) in the past month]	−0.26 (−0.64, 0.13)^*^	2.2 (1.3, 3.8)^**^	−0.37 (−0.72, −0.02)^**^	1.7 (0.9, 3.3)^*^		0.4
Cough as factor in multivariate model	−0.37 (−0.73, −0.01)^**^	2.1 (1.1, 4.0)^**^	−0.32 (−0.64, −0.01)^**^	1.7 (1.0, 3.1)		
Fever [vs. no fever episode(s) in the past month]	0.36 (−0.00, 0.72)^*^	0.7 (0.4, 1.4)	0.17 (−0.17, 0.52)	1.0 (0.5, 2.0)	0.09 (−0.40, 0.58)	0.9 (0.7, 1.2)
Fever as factor in multivariate model	0.53 (0.19, 0.88)^**^					
First born (vs. later born)	0.01 (−0.35, 0.36)	1.3 (0.7, 2.2)	−0.04 (−0.37, 0.28)	1.1 (0.6, 2.0)	0.21 (−0.26, 0.69)	0.8 (0.6, 1.0)^*^
First born as factor in multivariate model						0.8 (0.5, 1.2)
Low income (vs. >10,000 rupees/month)	−0.51 (−0.89, −0.14)^**^	1.5 (0.9, 2.7)^*^	−0.30 (−0.66, 0.05)^*^	1.4 (0.7, 2.6)	−0.37 (−0.87, 0.13)^*^	1.1 (0.9, 1.3)
Income as factor in multivariate model	−0.16 (−0.53, 0.21)	0.9 (0.4, 1.9)	0.08 (−0.26, 0.41)		0.35 (−0.14, 0.85)	
Maternal age (y)	0.02 (−0.02, 0.06)^***^	1.0 (0.9, 1.1)	0.03 (−0.01, 0.06)^*^	1.00 (0.93, 1.07)	0.07 (0.02, 0.12)^**^	1.00 (0.97, 1.02)
Maternal age as factor in multivariate model	0.01 (−0.02, 0.05)		0.02 (0.01, 0.06)		0.06 (0.01, 0.11)^**^	
Maternal height (cm)	0.06 (0.03, 0.08)^***^	0.9 (0. 9, 1.0)^**^	0.05 (0.02, 0.07)^**^	1.0 (0.9, 1.0)^**^	0.05 (0.01, 0.08)^**^	1.0 (1.0, 1.00)^*^
Maternal height as factor in multivariate model	0.04 (0.01, 0.07)^**^	0.9 (0.9, 1.0)^**^	0.03 (0.01, 0.06)^**^	1.0 (0.9, 1.1)	0.04 (0.01, 0.08)^**^	1.0 (1.0, 1.0)
Maternal height <150 cm (vs. ≥150 cm)	−0.58 (−0.92, −0.24)^**^	2.4 (1.4, 4.2)^**^	−0.52 (−0.84, −0.21)^**^	2.3 (1.2, 4.3)^**^	−0.30 (−0.76, 0.16)^*^	1.1 (0.9, 1.4)
Short maternal height as factor in multivariate model	0.9 (0.6, 1.6)	1.3 (0.4, 3.9)	0.9 (0.5, 1.4)	1.9 (1.0, 3.7)^**^	0.42 (−0.27, 1.11)	
Maternal education ≤8th standard (vs. >8th)	−0.54 (−0.91, −0.16)^**^	1.7 (1.0, 2.9)^*^	−0.49 (−0.83, −0.14)^**^	2.3 (1.3, 4.2)^**^	−0.10 (−0.61, 0.41)	1.0 (0.8, 1.2)
Maternal education as factor in multivariate model	−0.41 (−0.75, −0.06)^**^	1.5 (0.7, 2.8)	−0.35 (−0.00, −0.05)^**^	1.5 (0.8, 2.9)		

*Correlates refer to child unless noted by “maternal”; LAZ, length-for-age Z-score; WAZ, weight-for-age Z-score; WLZ, weight-for-length Z-score; Low income defined as <10,000 Indian Rupees (INR) per month (142% of the poverty line) (28); cough, fever, and diarrhea as reported occurring today or within the past 4 weeks*.

* *P ≤ 0.20*;

** *P <0.05*;

*** *P <0.0001*;

% *Multivariate model retained only 1 correlate signifcant at p <0.05*.

Among all children, multivariable models revealed that only increasing maternal height was associated a lower risk of underweight [0.94 (0.91, 0.97)], which was also associated with a higher WAZ; low birthweight was associated with a −0.69 (95% CI: −0.96, −0.42) lower WAZ (Table 2). In males and in females, low birthweight and decreasing maternal height were associated with lower WAZ. A higher risk of underweight [RR: 1.9 (95% CI: 1.0, 3.7)] when maternal height was <150 cm was observed among female children, as well as lower WAZ among female children who were reported to have at least 1 coughing episode within the past month (Table 4).

In multivariate models, low birthweight was associated with a greater risk of wasting [RR: 2.9 (95% CI: 1.5, 5.6)] and lower WLZ [0.62 (95% CI: −0.88, −0.35)] (Table 2). A higher risk of wasting [RR: 2.8 (95% CI: 1.3, 6.1)] was associated with report of at least one episode of fever within the past month.

Being male, having a low birthweight, and having had at last 1 diarrhea episode were associated with lower hemoglobin concentrations, while being firstborn and older maternal age were associated with higher hemoglobin concentrations (Table 2). Analyses carried out in male or female children alone showed different correlates by sex: Report of at least one diarrheal episode within the past month was associated with a 40% (20, 60%) greater risk of anemia and 0.70 (95% CI: 0.05, 1.35) g/dL lower hemoglobin among female children. Among males, birth order = 1 was associated with 20% (95% CI: 0, 70%) lower risk of anemia [and a 0.38 (95% CI: 0.1.13, 0.12) g/dL lower hemoglobin concentration] in addition to older maternal age, which was associated with a 3% (95% CI: 0, 5%) lower risk of anemia per additional year.

## Discussion

The overall purpose of this study was to describe the prevalence of undernutrition in a specific understudied vulnerable population: 10–18 month-old children in urban slums of Mumbai. We also describe the correlates of undernutrition in this context and compare and contrast the results to other settings in the following text. In the present study, we found a high prevalence of stunting, underweight and anemia and to a lesser degree, wasting among children residing in urban slums. Overall, a higher risk of stunting and lower LAZ score was observed for several correlates including male sex, low birthweight, shorter maternal height, and less maternal education. The risk of underweight and lower WAZ was associated with shorter maternal height and low birthweight, which was similar among both male and female children. Wasting and lower WLZ was associated with low birthweight and report of fever. Lower hemoglobin concentrations were associated with male sex and low birthweight, and report of diarrhea was associated with both lower hemoglobin concentrations and anemia. Differences by sex were observed, including that low birthweight and report of illness (fever and cough) were associated with stunting and low LAZ in females but not males; similarly, report of cough was associated with lower WAZ among females but not males. The association between correlates and hemoglobin concentration also varied by sex; birth order and maternal age were associated with anemia and hemoglobin only among males, while the association between diarrhea and hemoglobin was observed only among females.

The prevalence of stunting, underweight and anemia among young children in urban slums of India in this study were higher and that of wasting was lower that Mumbai's urban population (35). Previous studies have shown that children in urban slums suffer a higher proportion of undernutrition compared to non-slum areas (36). For example, in a 2013 study in Bangladesh, children under 5 living in urban slums had nearly double the prevalence of stunting and wasting compared to those living in urban non-slum areas (37).

A 2013 review (8) assessed 38 studies which examined risk factors of undernutrition among children in urban slum settings around the world and concluded that we need to understand these risk factors better to conduct more effective nutritional intervention trials in these vulnerable populations. From these 38 studies, the six most-reported risk factors in previous studies included (in descending order) maternal education status, age of the child, sex of the child, household income, report of illness, and family size (8). We compare and contrast the results of the present study and previous literature in the following text, focusing on studies carried out in urban slums of India among children under 5 years. Of note, most of these studies only analyzed differences in the prevalence of undernutrition among various risk factors and did not assess the differences in risk associated with each correlate in multivariate modeling, which our study adds to the literature.

Our findings on differences in undernutrition outcomes by sex corroborates the review by Goudet et al. (8), who reported eight studies conducted in urban slum populations that showed more male children were malnourished than female children; additionally, a recent longitudinal study in Bangladesh (38) and research in the slums of Aligarh city, Uttar Pradesh (39) support this result. However, contrasting findings were reported in urban slums of Jamnagar city (40) and Vadodara city (41), Gujarat; Srinagar District, Jammu and Kashmir (42); and Burdwan town, West Bengal (43). The proportion of undernutrition did not differ by in slums in Bankura (44), in Patiala, Punjab (45) or in Kolkata, West Bengal (46). However, these studies studied varying age groups, and as Bandyopadhyay et al. showed, the gender differential may depend on the age range studied (46) and the exact outcome studied: for example, females had a higher risk of underweight than males, but not for stunting or wasting (47). Possible biological mechanisms explaining higher risk for undernutrition among males include that male infants have increased metabolic requirements and thereby induce a greater nutrient deficit, manifesting as stunting, underweight, or wasting (48); that the greater number of males at conception causes male children to be more vulnerable according to evolutionary theory favoring a sex ratio of 1.0 (49); and that females have an immunological advantage over males (14, 50). Elucidating and reporting differences by sex is of importance for intervention studies (51) in order to better understand and programmatically address each population's particular needs, particularly in the realm of nutrition and susceptibility to diseases.

The present analysis showed that age was not a significant correlate for undernutrition, though this may be due to the narrower age range analyzed (10–18 months), compared to age ranges from 0 to 5 or even 0–10 years in previous studies. In previous studies, child's older age was associated with a higher risk for stunting and underweight while wasting was associated with younger age groups (starting at 6 months) (8). Delayed complementary feeding, which is associated with poor weaning practices, may be one factor to explain the effect of age on malnutrition (41). Low birthweight was associated with undernutrition in this study, and has been shown to be a significant correlate of poor growth outcomes in previous literature (47, 52–60), likely due to nutrition deficit accumulation over time (61–64). Meeting nutritional requirements before and during pregnancy may help improve weight at birth and minimize the need for catch up growth, as summarized in a recent systematic review (65). However, the studies carried out in Indian urban slums assessing the prevalence of poor growth have used heterogeneous definitions of undernutrition (32, 66–68) and analyzed different age ranges of children, limiting comparability (39–47, 69–75).

Child's illness, measured as the report of at least 1 episode of coughing, fever, or diarrhea in the past 1 month (shortened to either “cough,” “fever,” or “diarrhea,” respectively, in the following text for brevity) was the main correlate studied in the present study. Eight of the 38 previous studies (8) included a measure of illness in analysis, and diarrhea was the illness most commonly associated with moderate and severe undernutrition. However, only two of the 8 studies were done in Indian urban slums among children under 5 years of age (76, 77). These studies reported an association between more episodes of diarrhea in the past 15 days and negative deviance in undernutrition (76), and that children with moderate or severe pneumonia had a higher prevalence of severe undernutrition (77).

Maternal factors, including height, education, and age, were among correlates associated with child undernutrition. Our findings regarding maternal height are similar to previous research (53–55, 57, 61, 78–86), and supports the aforementioned intergenerational cycle hypothesis such that shorter mothers give birth to smaller offspring (64). In line with this hypothesis, a previous study showed that children with low birthweight whose mothers were <150 cm tall had a 2-fold higher odds of stunting, compared to low-birthweight children whose mothers were at least 150 cm tall (53). Another study showed that adolescent girls who were stunted in childhood and whose mothers were <145 cm tall were more likely to be stunted in adolescence, thereby increasing the chance of short stature during adulthood (87). Regarding maternal height and hemoglobin, there is little additional evidence assessing shorter maternal height in association with anemia among children beyond one study. Further exploration of the association between maternal stature and child's hemoglobin concentration is warranted (82). Inclusion of maternal education and/or literacy level represented the most-reported risk factor, being included in 12 out of 38 studies reviewed (8). Generally, higher maternal education was associated with lower risk of undernutrition, globally (8) and within Indian urban slums among infants under 5 (40, 44, 45). Some studies not included in the review and more recent studies among children under 5 years of age living in urban slums (71, 74–76, 88) also showed a significant association between maternal education and poor growth. None of these later studies analyzed the association between years of maternal education attained and undernutrition using multivariate modeling among young children in urban slums of India.

Family correlates such as household income and birth order were analyzed in association with undernutrition. Household income was not a significant correlate for undernutrition in multivariate analyses; this may have been due to the lower-resolution nature of this data, as the lowest income group (0–10,000 rupees per month) data available was equivalent to 142% of the urban India poverty line (28) and may have masked an association between an income lower than 10,000 rupees and undernutrition. However, previous studies reported low household income as a significant risk factor for undernutrition, and this is reflected globally as well as in India-specific contexts, as detailed in the review (8). Birth order was our variable for approximating family size. Our finding that males benefitting from firstborn status (unlike females) is in line with findings from the review (8), which included either family size or birth order, and showed that larger family size was a predictor of undernutrition (44, 76). None of these studies assessed family size as a correlate for anemia. The association between smaller family size, earlier birth order/fewer siblings and better nutrition status has been supported in other studies of urban India as well (47, 72, 74), which may be explained by the fact that the firstborn child of either gender may be cared for more, and that second (and later) born children suffer discrimination when there is either already a son in the family or if the family does not produce a son (74).

## Limitations

Some limitations of this study may include the measurement of our correlates, potential risk of sampling bias, and generalizability. For example, the correlate monthly family income was collected categorically and not continuously, leading to lower resolution of the lowest income bracket of <10,000 rupees, which most families reported; it would be interesting to analyze the per-capita monthly income to understand this correlate more fully. Further, we were able to collect only binary data on morbidity; data on the number of episodes of an illness would have added greater understanding to our study. The narrow age range studied limits the inferences we can make about the contribution of age to undernutrition. We were not able to collect ethnicity data from the majority of participants, limiting our ability to determine possible variation by village or region of origin. Additionally, a recent review showed that measuring the prevalence of stunting may not be the best indicator of child linear growth, as the definition of stunting is not biologically-based and may underestimate the prevalence of linear growth impairment (89). Because only participants who could come to the study center for profiling could be included in this analysis, there is a risk for sampling bias. We note that external generalizability to children living in urban slums outside of India is limited, however, given that this analysis included children from 20 different urban slum areas, of varying socioeconomic status, we believe that the findings of this study may be generalizable to urban slums within India, particularly for children in their 2nd year of life.

## Conclusions

This is the first study to investigate both the prevalence of poor growth and anemia as well as the impact of commonly-reported correlates on the likelihood of undernutrition among young children living in Mumbai urban slums. Consistent correlates for poor growth and anemia included low maternal height, low birthweight, being male, being firstborn, and report of illness in this study. Child undernutrition remains a persistent issue around the world yet remains understudied particularly in vulnerable settings such as urban slums, which have a high prevalence of undernutrition as shown by this study. The data presented here suggests that the effectiveness as well as reach of nutritional supplement programs such as Integrated Child Development Services may need to be reexamined particularly in urban slums among children in their second year of life. Other correlates of undernutrition in children suggested by other studies, such as circulating metabolites (90, 91) the gut microbiome (92–94), blood micronutrient concentrations (95), and immune function (96) require further exploration.

## Ethics Statement

The protocol was reviewed and approved by the Inter Systems Biomedical Ethical Committee (ISBEC, Mumbai, Maharashtra, India), St. John's Research Institute (SJRI) Institutional Ethics Committee (IEC), and the Institutional Review Board (IRB) at Cornell University. In addition, permissions to conduct the study were obtained from the Health Ministry Screening Committee of India (Indian Council of Medical Research). Informed consent was obtained from all caregivers in an audio/visual format per Indian Government guidelines prior to screening and enrollment in the parent trial.

## Author Contributions

SM, JH, JF, SU, PG, RP, and AK designed research (project conception, development of overall research plan, and study oversight). SH, SV, HC, AT, and VT conducted research (hands-on conduct of the experiments and data collection). SH and SM performed statistical analysis. SH wrote the first draft of the manuscript. SM had primary responsibility for final content.

### Conflict of Interest Statement

SM is an unpaid board member for and has an equity stake in a diagnostic start up focused on developing point-of-care assays for nutritional status informed by his research as a faculty member at Cornell University. The remaining authors declare that the research was conducted in the absence of any commercial or financial relationships that could be construed as a potential conflict of interest.
